# Aging induces B cell defects and decreased antibody responses to influenza infection and vaccination

**DOI:** 10.1186/s12979-020-00210-z

**Published:** 2020-11-19

**Authors:** Daniela Frasca, Bonnie B. Blomberg

**Affiliations:** Department of Microbiology and Immunology and Sylvester Comprehensive Cancer Center, University of Miami Miller School of Medicine, RMSB 3146A, 1600 NW 10th Ave, Miami, FL 33136 USA

**Keywords:** Aging, B cells, Antibodies, Influenza infection, Influenza vaccination

## Abstract

**Background:**

Aging is characterized by a progressive decline in the capacity of the immune system to fight influenza virus infection and to respond to vaccination. Among the several factors involved, in addition to increased frailty and high-risk conditions, the age-associated decrease in cellular and humoral immune responses plays a relevant role. This is in large part due to inflammaging, the chronic low-grade inflammatory status of the elderly, associated with intrinsic inflammation of the immune cells and decreased immune function.

**Results:**

Aging is usually associated with reduced influenza virus-specific and influenza vaccine-specific antibody responses but some elderly individuals with higher pre-exposure antibody titers, due to a previous infection or vaccination, have less probability to get infected. Examples of this exception are the elderly individuals infected during the 2009 pandemic season who made antibodies with broader epitope recognition and higher avidity than those made by younger individuals. Several studies have allowed the identification of B cell intrinsic defects accounting for sub-optimal antibody responses of elderly individuals. These defects include 1) reduced class switch recombination, responsible for the generation of a secondary response of class switched antibodies, 2) reduced de novo somatic hypermutation of the antibody variable region, 3) reduced binding and neutralization capacity, as well as binding specificity, of the secreted antibodies, 4) increased epigenetic modifications that are associated with lower antibody responses, 5) increased frequencies of inflammatory B cell subsets, and 6) shorter telomeres.

**Conclusions:**

Although influenza vaccination represents the most effective way to prevent influenza infection, vaccines with greater immunogenicity are needed to improve the response of elderly individuals. Recent advances in technology have made possible a broad approach to better understand the age-associated changes in immune cells, needed to design tailored vaccines and effective therapeutic strategies that will be able to improve the immune response of vulnerable individuals.

## Introduction

Aging is characterized by a progressive and significant decline in immune function, called immunosenescence. Both innate and adaptive, and within the latter, cellular and humoral immune responses are decreased by aging, leading to increased frequency and severity of infectious diseases and reduced responses to vaccination [1, 2]. Hospitalization following infection is more common in the elderly than in younger individuals and is a major contributor to the development of disability [1].

Aging is associated with increased chronic low-grade systemic inflammation, known as inflammaging [3], which represents a significant risk factor for morbidity and mortality of the elderly as it is implicated in the pathogenesis of several debilitating chronic diseases such as Type-2 Diabetes Mellitus, osteoporosis, Alzheimer’s disease, cancer, rheumatoid arthritis, chronic obstructive pulmonary disease, and coronary heart disease, among others. Inflammaging induces intrinsic inflammation in immune cells leading to decreased protective responses against infections and decreased vaccine responses [4–6]. Inflammaging also contributes to metabolic dysfunction and development of insulin resistance (IR) [7].

Inflammaging is driven by several factors, such as single nucleotide polymorphisms in the promoter regions of pro- and anti-inflammatory genes, chronic stimulation of immune cells with pathogens, changes in the composition of the gut microbiota, and cellular senescence [8]. Another mechanism for fueling inflammaging has recently been proposed and called “garbaging”. Garbaging indicates the age-related increase in the generation of cell-derived products of damaged or dead cells (endogenous, misplaced or altered molecules). The disposal of these products through the proteasome is significantly decreased by aging, leading to the activation of innate immune cells and consequent release of pro-inflammatory mediators [9].

Decreased T cell function has been considered for many years the only contributor to immunosenescence. However, age-related defects in other components of the immune system also occur and contribute to the increased frequency and severity of infectious diseases in the elderly. In this review, we primarily focus on human B cell defects with aging, and how these contribute to reduced responses to influenza infection and vaccination. A thorough analysis of T cell defects contributing to the decreased response to influenza infection and vaccination of the elderly has recently been published [10]. Also, see the articles covering other immune cell defects in this special issue compilation.

## Aging is associated with increased rates of influenza infection

Influenza infection is a global major public health concern. Worldwide, there are millions of hospitalizations and thousands of deaths reported each year due to seasonal epidemics. The establishment of a virologic surveillance network for influenza is challenging for many countries because of the requirements to collect, analyze, and report local viral surveillance data for large numbers of individuals. For this reason, most of the available data have been obtained in high-income countries. Many developing countries have recently improved their influenza surveillance and have developed country-specific influenza-associated mortality estimates, which are generally higher than those calculated by the World Health Organization, suggesting that current global influenza death estimates might underestimate the global mortality burden of influenza. A recent study has calculated country-specific influenza-associated respiratory excess mortality rates (EMR) in 33 low-income and high-income countries around the world, from 1999 to 2015. The results have shown that EMR ranged from 0.1 to 6.4/100,000 individuals for people younger than 65 years, 2.9 to 44/100,000 individuals for people aged 65–74 years, and 17.9 to 223.5/100,000 for people older than 75 years [11].

The Centers for Disease Control and Prevention monitor laboratory-confirmed influenza infections and associated hospitalizations in the U.S. through the Influenza Hospitalization Surveillance Network (FluSurv-NET) (https://www.cdc.gov/mmwr/volumes/68/wr/mm6824a3.htm). Data from the 2018–2019 season have confirmed previous year data and have shown that hospitalization rates were higher among individuals aged ≥65 years. Almost all (95%) of the hospitalizations were due to influenza A virus, only 4% to influenza B virus and 1% to influenza A and B virus co-infection. Among influenza A virus infections, about 50% were with the A/H3N2, a strain known to lead to heavy seasonal epidemics [12]. A/H3N2 is also the most common virus affecting elderly individuals, leading to the highest hospitalization rates and death [13].

Also, data from Europe collected during the 2018–2019 season (https://www.ecdc.europa.eu/en/publications-data/seasonal-influenza-annual-epidemiological-report-2018-2019) have shown the the vast majority of influenza viruses detected were type A, both subtypes (A/H1N1pdm09 and A/H3N2), with different distributions of A subtypes among different European countries. Very low numbers of type B viruses (B/Yamagata and B/Victoria) were detected in the 2018–2019 season. ICU cases were mainly aged 65 years and older, with most hospitalizations being due to influenza A infections.

In addition to advanced age and the age-related decrease in immune function, other factors increase the risk of influenza infection. These include chronic diseases, obesity, metabolic diseases, immunosuppression, and neuromuscular disorders [14]. Frailty, a measure of health, physical function and physiologic reserve, also strongly predictive of health outcomes, is another risk factor for influenza infection [15]. Once infected with influenza, elderly individuals are also at increased risk to get other infections with viruses that cause severe respiratory tract (RT) infections, such as respiratory syncytial virus (RSV), parainfluenza virus, rhinovirus, enterovirus, and coronavirus. Influenza infection affects both the upper and the lower respiratory systems, inducing common cold, sinusitis, tonsillitis, pharyngitis and laryngitis in the upper RT, and pneumonia, bronchitis and tuberculosis in the lower RT [16]. The infection with the influenza virus induces several changes that alter the proper function of the RT, as reviewed in [17]. Although not specifically demonstrated for infuenza infection, a dysfunctional RT may predispose elderly individuals to be also infected by other pathogens affecting the RT, and there is experimental evidence that in mice infected with influenza alveolar macrophages show sustained desensitization in response to TLR-mediated stimuli, characterized by a significant decrease in chemokine secretion leading to reduced recruitment of neutrophils and heightened bacterial load during secondary RT infections [18].

Since the last months of 2019, Severe Acute Respiratory Syndrome-Coronavirus 2 (SARS-CoV-2) had caused > 26 million cases of Coronavirus Disease 2019 (COVID-19) and > 870,000 deaths worldwide (as of September 4th, 2020, https://covid19.who.int). Early reports from China showed that frequency of co-infection with other RT viruses was limited in the whole population [19, 20]. Nevertheless, the Centers for Disease Control and Prevention endorsed testing for co-infection with other RT viruses, to increase the possibility to detect potential COVID-19 patients (https://www.cdc.gov/coronavirus/2019-ncov/hcp/clinical-criteria.html). The results from a study conducted by Stanford investigators showed higher rates of co-infection in the whole population, as compared to the initial results from China, with 20.7% of SARS-CoV-2 positive samples also positive for at least one RT virus. The most common co-infections were with rhinovirus/enterovirus (6.9%), RSV (5.2%) and non-SARS-CoV-2 Coronaviruses (4.3%). However, when the analysis was performed in young/adults versus elderly individuals, the frequency of elderly individuals co-infected with SARS-CoV-2 and another RT virus like influenza A virus was only 1% [21]. At the time that this review is written, there are still conflicting reports as to whether the infection with SARS-CoV-2 increases the rates of co-infection with other RT in the elderly population.

Co-infections with bacteria such as *S. pneumoniae* have also been reported as a significant cause of morbidity and mortality especially in the elderly and it has been shown that influenza viruses alter the lungs and favor the adherence, invasion and induction of disease by the bacterium. Several published results have clearly demonstrated that influenza virus-induced inflammation, and consequent immune dysfunction, reduce the ability of the host to clear pneumococcus [22]. Interestingly, very recently published observations have shown that the use of seasonal influenza and pneumococcal polysaccharide vaccines reduce COVID-19 mortality in older adults [23].

## Aging decreases antibody responses to the influenza virus

Both cellular and humoral adaptive immune responses play key roles in the clearance of the virus and in protection. The infection is initially controlled by an antibody response which allows time for cytotoxic T cell-mediated immune responses to develop. Antibodies bind to the hemagglutinin (HA) surface glycoprotein, that allows infection of the host cell, and neutralize the virus. Antibody titers measured by the HemAgglutination Inhibition (HAI) assay remain the gold standard correlate of protection against infection and represent the only measure of vaccine efficacy, although they do not provide clinical protection against influenza-associated complications.

In general, individuals with higher pre-exposure HAI titers have less probability to have a large/productive infection. The 2009 H1N1 pandemic unexpectedly had low morbidity and mortality in older people, likely because older people weve already been exposed to the 1918 pandemic A/H1N1 virus that gave rise to periodic seasonal strains that decreased in frequency only in the late 1950s. The 1918 and the 2009 pandemic A/H1N1 viruses had high levels of similarities, as indicated by the cross-neutralization and protection between the two viruses [24]. In a study conducted in 130 individuals of different ages (0–89 years old), infected with the 2009 H1N1 pandemic strain (H1N1pdm09), an in-depth evaluation of antibody responses was performed [25]. In addition to HAI titers, H1N1pdm09 whole genome fragment phage display libraries were used to evaluate antibody repertoires against internal genes, HA and neuraminidase, and to measure antibody affinity for antigenic domains within HA. Results showed that the infection of elderly individuals (≥70 years old) induced antibodies with broader epitope recognition than those induced in younger individuals (0–69 years old). Importantly, serum antibodies of elderly individuals had significantly higher avidity for the HA1 globular domain, but not for the conserved HA2 stalk, than those from younger individuals and lower antibody dissociation rates measured by surface plasmon resonance. This study provided the first evidence for a qualitatively superior antibody response in the elderly following H1N1pdm09 infection, suggestive of recall of long-term memory B cells or long-lived plasma cells [25].

## Aging decreases influenza vaccine-specific antibody responses

Despite reduced efficacy in elderly individuals, influenza vaccination is still the most effective way to prevent influenza infection and represents a cost-effective strategy, although it is clear that vaccines with greater immunogenicity are needed to improve the response of the elderly [26]. Vaccination has been shown to significantly reduce disease burden and influenza transmission within the community. Several vaccines are approved in the United States for seasonal influenza vaccination each year. Influenza vaccines require annual reformulation due to continuous viral evolution (antigenic drift and shift) which allows new human and non-human influenza viruses to infect human individuals. Annual influenza vaccination campaigns help individuals to make protective antibodies specific for the currently circulating strains. Influenza vaccines induce an antiviral response in B and T cells, and humoral and cellular immunity, respectively [27]. The antibody response to the vaccine represents the first line of protection from subsequent infection, with IgM antibodies providing initial protection, whereas class switched antibodies (IgA and IgG) neutralize newly replicating virus once infection has been established [28]. Although for a long time it has been believed that there is no pre-existing immunity to newly emerging influenza variants in humans [29, 30], it has been demonstrated that seasonal influenza vaccination can induce polyclonal heterosubtypic neutralizing antibodies which are cross-reactive with both the swine-origin H1N1pdm09 virus and the H5N1 avian virus [31].

Both the production and the duration of vaccine-specific high affinity protective antibodies decrease with age [32] and therefore yearly vaccination with the current influenza vaccine is strongly recommended for individuals ≥65 years of age to protect them from infection and associated complications. Young individuals respond better than elderly individuals to the first influenza vaccination. However, after repeated vaccinations with the same vaccine that increase vaccine-specific serum antibody titers [33], the difference between young and elderly individuals decreases, suggesting the importance of the history of an individual that includes previous vaccination and/or infection [34]. Influenza vaccine-specific antibodies do not persist year-round in older adults, and therefore alternative vaccination strategies providing better clinical benefits are needed [35]. It has indeed been shown that elderly individuals who have received the influenza vaccine can still be infected and can also get severe secondary complications such as hospitalization, catastrophic disability, exacerbation of underlying medical conditions and death [36–38], likely due to a compromised immune system in these individuals. Although vaccination rates have increased over the years, with > 60% vaccination rates in individuals ≥65 years of age in many countries, influenza is still a serious threat for the elderly, and it has been shown that in the U.S. ≥65 year old individuals account for 2/3 of the 200,000 influenza-related hospitalizations, independently of associated conditions. Moreover, the length of hospital stay for elderly individuals is almost 3- and 6-fold higher than middle age (50–64 year old) and younger individuals, respectively [10].

In addition to age, several causes determine the limited success of influenza vaccination among elderly adults. These include preexisting immunity, genetic polymorphisms, and the presence of chronic underlying conditions may compromise the influenza vaccine response [39, 40]. These factors are also relevant in younger individuals.

Systems vaccinology approaches have recently been successfully employed to evaluate and characterize innate and adaptive immune responses to the influenza vaccine. Systems vaccinology, employing systems biology approaches to identify molecular signatures at early post-vaccination time points (1–3 days), has provided a global picture of the immune response to the influenza vaccine. In a study conducted on 212 individuals of different ages recruited across 5 consecutive influenza vaccine seasons (2007–2011), we found, as expected, that the antibody response to the vaccine was negatively associated with age (not with gender and race) [41]. We performed a gene set enrichment analysis of genes correlated with the HAI response in each season and we found that signatures of innate immunity and plasmablasts were not only associated with, but also predicted, post-vaccination antibody titers with > 80% accuracy across multiple seasons. However, these signatures were not associated with the duration of the response. We also found that early post-vaccination signatures of inflammatory pathways in lymphocytes were positively associated (whereas those in monocytes were negatively associated) with post-vaccination antibody titers. The importance of these results relies in the identification of shared signatures of influenza vaccine-specific antibody responses across multiple seasons that can help to develop next-generation vaccines that can be highly protective.

Many other systems vaccinology studies have evaluated gene expression and cellular signatures correlated with protective responses to the influenza vaccine. Among these, a multicenter analysis has identified baseline transcriptional signatures that are significantly associated with vaccine-specific antibody responses in different cohorts recruited through the Human Immunology Project Consortium and the Center for Human Immunology, and applied a common quantitative metric to stratify relative vaccine responses in all cohorts. These validated signatures were specific to young vaccinees (< 35 years of age) but their effect sizes (strength of correlation with antibody responses) were also correlated in older participants. Some of these signatures have already been found in earlier studies and were primarily related to the frequencies of peripheral immune cell subsets, whereas others revealed the involvement of previously unreported genes and pathways, such as autophagy, anti-viral innate immunity, antigen presentation, BCR signaling [42]. In another study, baseline transcripts found significantly correlated with antibody responses were enriched for pattern recognition and interferon signaling [43], for age- and apoptosis-related gene module [44] or for lipid biosynthesis, also associated with sex and testosterone-dependent differences in the antibody response [45]. When the expression of markers of immunosenescence (age, frequency of T cell receptor excision circle, telomerase expression, frequencies of CD4+ CD28- and CD8+ CD28- T cells, CD4/CD8 T cell ratios) was evaluated, it was found that immunosenescence-related differences in gene expression, gene regulation, cytokine secretion, and immunologic changes were able to predict the lower response of older adults to seasonal influenza A/H1N1 vaccination [46]. Sex differential responses to the vaccine were also identified [47]. Gene expression patterns associated with vaccine-specific antibody responses were found to be different between young and old vaccinees also in another study in which the post-vaccination increase in the expression of KLRB1 (killer cell lectin-like receptor B1) was positively associated with vaccine-specific antibody responses in young but not in older adults [48]. A meta-analysis combined publicly available influenza microarray data with the aim of identifying the effects of disease (influenza infection, influenza vaccination, controls), age and sex on gene expression. Results have identified around 1000 differentially expressed genes when results were filtered by disease factor, and gene enrichment analysis, with statistically significant disease-age interactions. Genes included innate immune response, viral process, defense response to virus, hematopoietic cell lineage and NF-kB signaling pathway [49]. A mitochondrial signature of young and old influenza vaccine responders has identified genes and proteins controlling mitochondrial biogenesis and pathways of oxidative phosphorylation [50]. It was found that these pathways and genes involved in cellular respiration, heme biosynthesis, mitochondrial DNA transcription and regulation were up-regulated in young and, to a significantly lower extent, in older vaccine responders. This study was the first genome-wide transcriptional analysis of age-associated metabolic measures performed before and after influenza vaccination, showing the crucial role of mitochondrial pathways in influenza vaccine responses.

## Mechanisms for reduced influenza vaccine-specific antibody responses in the elderly

Age-related changes in the function of T cells [32, 51–53], NK cells [54, 55] and antigen-presenting cells [56, 57] have been shown to contribute to the reduced influenza vaccine-specific response in the elderly and increased frequency and severity of influenza infection. We have previously identified intrinsic B cell defects with age also accounting for the sub-optimal influenza vaccine response of elderly individuals [5, 58–63]. We will summarize below published results on age-related changes in B cells, focusing on the cellular and molecular mechanisms involved in the regulation of influenza vaccine responses.

### Age effects on class switch recombination (CSR) and somatic hypermutation (SHM)

The B cell intrinsic age-related defects in class switch recombination (CSR) and somatic hypermutation (SHM) lead to sub-optimal antibody responses to the influenza vaccine. CSR and SHM are two processes needed for the generation of secondary class switched high affinity antibodies [64]. Reduced CSR and SHM with age in mice and humans are due to decreased expression of activation-induced cytidine deaminase (AID) and of E47 [65], encoded by the E2A gene, a key transcription factor regulating AID [66]. Also PAX5, another key regulator of CSR and antibody production [67, 68], has recently been shown to be reduced in mature B cells from elderly as compared to young individuals and to be associated with the expansion of pro-inflammatory B cell subsets [69], which are unable to respond to influenza vaccination [70].

We have measured the B cell antibody response to the influenza vaccine in vivo and in vitro, by HAI and by AID mRNA expression by qPCR after in vitro restimulation with the vaccine, respectively. Both responses are decreased with age and are significantly correlated, supporting our initial hypothesis that the in vitro AID response recapitulates what has occurred in vivo in the germinal center in the generation of memory B cells. After 2009, the H1N1pdm09 has been repeated in the vaccine preparation for several years, leading to higher levels of seroprotection in vaccinated individuals, suggesting that the memory B cells to H1N1 can be stimulated in elderly individuals [71, 72].

We have evaluated if AID was also involved in antibody affinity maturation in young and elderly individuals vaccinated with the H1N1pdm09 vaccine. We found that in both young and elderly individuals the fold-increase in AID after vaccination correlated with fold-increase in polyclonal antibody affinity to the globular HA1 (but not to the conserved HA2) antigen of the H1N1pdm09. Affinity maturation to the HA1 domain was observed after vaccination only in young individuals. In the elderly, high affinity antibodies specific for the HA1 domain were already present before vaccination and the change in antibody affinity was almost undiscernible, suggesting a previous vaccination or infection with highly conserved H1N1pdm09 determinants which were able to generate an optimal memory response. These findings were the first to demonstrate a strong correlation between AID induction and in vivo antibody affinity maturation in humans [72].

### Age effects on the generation of memory B cells

Despite the age-related decrease in AID, influenza vaccine-specific memory B cells are maintained in the elderly after repeated vaccination with a vaccine containing the same H1N1pdm09, but the fold-increase in HAI titers after vaccination is lower in the elderly although most of them are seroprotected, suggesting that low seroconversion in the elderly may be mainly due to cell intrinsic defects in the differentiation of B memory cells to plasmablasts/antibody-secreting cells (ASCs) [71]. The analysis of the antibodies secreted from single plasmablasts 7 days after influenza vaccination has indeed shown an age-related decrease not only in the number of vaccine-specific plasmablasts, but also in the quantity of antibodies made by these cells [73]. One possible explanation for similar memory B cell responses in young and elderly individuals is that B cells carrying a switched IgG receptor could be positively selected, and proliferate in response to repeated vaccinations, in elderly individuals similar to what has been observed in aged mice [74]. Influenza vaccine-specific memory B cells represent an important pool of cells able to mount a rapid secondary immune response and contribute to both short-term and long-term immunity. These data altogether suggest that at least in the case of influenza vaccine, due to intrinsic age-related impairment in the differentiation of ASCs, regular booster vaccinations are highly recommended to induce seroprotective titers and protect the elderly from influenza infection.

### Age effects on B cell repertoire

Although there is an age-related decrease in the number of vaccine-specific plasmablasts, as well as in the quantity of antibodies made by these cells, the avidity of vaccine-specific antibodies and the affinity of recombinant monoclonal antibodies obtained from single plasmablasts seem to be not significantly different in young and elderly individuals [73]. In agreement with these findings, the analysis of the clonal structure and mutation distribution of B cell repertoires has shown that elderly individuals had decreased numbers of lineages but increased pre-vaccination mutation load in their repertoire, suggesting previous infections or vaccinations [75]. Moreover, some of these individuals have an oligoclonal repertoire in which the diversity of the lineages is greatly reduced as compared to young individuals, consistent with earlier reports on contraction of B cell repertoires in the elderly [76]. The spectratype analysis and high-throughput sequencing, the B cell repertoire of elderly individuals has shown evidence of non-specific clonal expansions in the absence of challenge, with this loss of specific B cell diversity being correlated with poor health [77]. Another study, also showing comparable blood frequencies of plasmablasts in young and elderly individuals, showed similar V/D/J usage in young and elderly vaccine responders. However, recombinant antibodies generated from plasmablasts of elderly vaccine responders bound a broader number of HA epitopes, indicating an increased breadth of binding across influenza strains [78].

High-throughput DNA sequencing of B cell receptor IgH gene rearrangements occurring in vaccinated young and elderly individuals has shown that V/D/J usage is similar in B cells from young and elderly individuals. Moreover, because seropositivity with latent viruses such as cytomegalovirus (CMV) or Epstein-Barr virus (EBV) may change the B cell repertoire, CMV-positive and EBV-positive individuals of different ages were evaluated. It was found that infection with both CMV and EBV alters the B cell repertoire, regardless of the individual’s age, with CMV infection being correlated with the proportion of V_H_ mutations in IgM and IgG antibodies and EBV infection being correlated with the presence of persistent clonal B cell expansions [79].

An age-related decrease in the accumulation of de novo mutations in antibodies induced by influenza vaccination was reported and found to be associated with reduced binding capacity of the antibodies to the viral glycoprotein HA and reduced neutralization capacity. These antibodies were also targeting non-HA epitopes [80]. Antibodies with de novo mutations are those able to adapt to drifted and shifted influenza viruses and are therefore highly protective. It has been shown that antibodies from elderly individuals predominantly target highly conserved (but not potent) epitopes outside the HA receptor-binding site, and generate less protection [80]. These results have suggested that elderly individuals rely on cross-reactive memory B cells generated early in life for protection.

### Effects of age-associated epigenetic modifications on influenza vaccine responses

A recent study of baseline whole genome DNA methylation in peripheral blood mononuclear cells of responders and non-responders to the influenza vaccine of different ages has identified possible age-related DNA methylation contributors to vaccine responsiveness [81]. This study discovered 142 differentially methylated CpG sites in young and 305 in elderly individuals, respectively, suggesting a larger epigenetic remodeling with aging. Interestingly, some of the differentially methylated probes mapped in genes involved in immunosenescence (CD40) [82] and in innate immune responses during viral infections (CXCL16, ULK1, BCL11B) [83–85]. Another study on epigenetic and transcriptomic profiles and humoral immune responses in young and elderly recipients of the influenza vaccine has also shown associations between DNA methylation and gene expression and has revealed a system-wide regulation of immune relevant functions that play crucial roles in regulating the participant’s capacity to respond to vaccination. Results have identified a group of CpGs that are associated with lower or higher antibody responses, when coordinately hypo-methylated or hyper-methylated, respectively. Interestingly, CpGs that individually predict antibody responses are enriched for *FOXP2* transcription factor binding sites, with the most robust associations involving differential methylation of genes associated with immunity and immunosenescence [86]. These studies, leading to the identification of differentially methylated CpG sites between responders and non-responders to the influenza vaccine, represent an important step to gain knowledge on potential targets of immunosenescence that may help to design better vaccines for the elderly.

### Age effects on B cell subsets

Another mechanism that can account for the reduced B cell response to the influenza vaccine in the elderly is the well-defined redistribution of peripheral B cell subsets that occurs during aging, with a significant expansion of a subset of B cells that is the most pro-inflammatory, called Double Negative (DN) B cells, as we [87, 88] and others [89, 90] have reported. These cells, also called late/exhausted memory or tissue-like memory B cells, have been identified as CD19 + CD27-IgD- and have been shown to be increased in the blood of patients with autoimmune [89, 91, 92] and infectious diseases [93–95], and it has been suggested that they may expand in vivo in the presence of autoantigens or pathogen-derived antigens, in the context of a favorable inflammatory microenvironment, leading to the production of autoimmune or protective antibodies, respectively. Their frequencies in blood are negatively associated with the serum response to the influenza vaccine [87], due to the fact that they secrete large amounts of pro-inflammatory cytokines making them refractory to further stimulation. DN B cells do not proliferate and do not make antibodies to influenza antigens, but they secrete antibodies with autoimmune reactivity, in agreement with their membrane phenotype (CD95 + CD21-CD11c+) and their spontaneous expression of the transcription factor T-bet, both characteristics of autoimmune B cells [70].

DN B cells are the human equivalent of the subset of mouse splenic B cells called Age-associated B Cells (ABC) [96, 97]. Both DN B cells and ABCs are generated from conventional mature B cell subsets (naïve in humans, follicular B cells in mice) after in vivo or in vitro stimulation with the endosomal nucleic acid-sensing Toll-like receptors TLR7 or TLR9, alone or together with BCR cross-linking, demonstrating that BCR is also an active signaling system in these subsets. TLR engagement in the presence of the cytokines IL-21 and IFN-γ regulate T-bet expression, whereas in the presence of IL-21 alone promotes CD11c expression independently of T-bet [98]. T-bet+ ABCs carry high frequencies of somatically mutated Ig, suggesting that they originate during T-dependent B cell responses [99]. T-bet+ ABCs appear and persist indefinitely after influenza infection in mice [98, 99]. These cells represent the spleen-resident population of memory B cells esponsible for the secretion of HA stalk-specific IgG2c antibodies and of durable neutralizing antibodies [100]. Previous results from a different group have also demonstrated the specificity of mouse ABCs for a live influenza virus (A/PR8/34) and these influenza-specific ABCs differentiate into antibody-secreting cells, some of which home to the bone marrow and to the lungs, and persist for long periods of time after infection (> 4 weeks), suggesting their role in providing significant protection [101]. Human T-bet+ B cells also have been shown to mediate influenza-specific humoral memory [100]. Similar to mouse T-bet+ ABCs, they have an activated phenotype, they are spleen-resident and secrete HA-specific IgG1 (the equivalent of mouse IgG2c) antibodies recognizing H1 or H3 viral strains.

DN B cells increase under obesity conditions, reaching significantly high frequencies (up to 50% of total B cells) in the obese subcutaneous adipose tissue (AT), where they also secrete large amounts of autoimmune antibodies specific for adipocyte-derived proteins, supporting the hypothesis that DN B cells would generate suboptimal immune responses by circulating to peripheral lymphoid organs. The AT is the largest organ in humans and therefore age-related changes in AT composition and function, as well as in AT metabolism, may lead to significant systemic changes which in turn may accelerate and exacerbate the aging process. Fat mass increases with age in humans [102, 103] and this is associated with increased inflammaging [3], which contributes to metabolic dysfunction and development of IR which also increases with age. Moreover, an age-associated increase in the ectopic deposit of triglycerides in several tissues (liver, muscle, heart, pancreas, kidney) [104–108] and in blood vessels [109] occurs, and this is associated with the development and/or progression of age-associated diseases.

### Age effects on telomeres

B cells from elderly individuals with protective titers to the influenza vaccine had significantly longer telomeres than those with poor antibody responses [110], suggesting a role of telomeres in supporting the expansion of vaccine-specific B cells by preserving their replicative lifespan. Telomeres are specialized terminal chromosomal structures that consist of tandem hexanucleotide repeats (TTAGGG)n and telomere binding proteins with the function of maintaining the integrity of chromosomes during chromosomal replication [111]. Based on these results, it is likely that the age-related decrease in telomeres can be another mechanism explaining why B cells from some elderly individuals cannot proliferate and differentiate in response to the influenza vaccine.

## Conclusions

Aging decreases antibody responses to the influenza virus and to the influenza vaccine. The decreased antibody response to the influenza virus is best exemplified by the decreased influenza vaccine efficacy, and several studies have identified the molecular mechanisms responsible for sub-optimal antibody responses of elderly individuals. Although influenza vaccination represents the most effective way to prevent influenza infection, vaccines with greater immunogenicity are still needed now to overcome the immune defects and improve the response of elderly individuals. Systems vaccinology approaches have better characterized the age-associated immune defects which can be used to design tailored vaccines and better therapeutic strategies to induce stronger humoral and cell-mediated immunity in vulnerable individuals like the elderly.

## Data Availability

Not applicable.

## Abbreviations

AID: Activation-induced cytidine deaminase
ASCs: Antibody-secreting cells
CMV: Cytomegalovirus
CSR: Class switch recombination
EMR: Excess mortality rates
HA: Hemagglutinin
HAI: Hemagglutination inhibition
RT: Respiratory tract
SHM: Somatic hypermutation
